# Research progress of artificial intelligence in moyamoya disease

**DOI:** 10.3389/fneur.2025.1581338

**Published:** 2025-05-16

**Authors:** Huimin Huang, Ning Zheng, Lei Feng, Shuo Shao

**Affiliations:** ^1^Radiological Medical College, Shandong First Medical University and Shandong Academy of Medical Sciences, Jinan, China; ^2^Department of Radiology, Jining No. 1 People's Hospital Affiliated to Shandong First Medical University, Jining, China; ^3^Department of Neurosurgery, Jining NO.1 People’s Hospital Affiliated to Shandong First Medical University and Shandong Academy of Medical Sciences, Shandong Provincial Key Medical and Health Laboratory of Neuroinjury and Repair, Jining, Shandong, China

**Keywords:** artificial intelligence, moyamoya disease, diagnosis, risk factors, treatment, basic research

## Abstract

Moyamoya disease (MMD), a chronic, progressive cerebrovascular disorder of unknown etiology, presents significant diagnostic and therapeutic challenges in clinical practice. Conventional diagnostic methods rely on physicians’ experience and have limitations in disease prediction, risk assessment, and treatment decisions. The advancement of artificial intelligence (AI) technologies has created new opportunities for research on MMD. This review summarizes recent advances in AI applications for MMD, including diagnosis, risk factor analysis, treatment planning, outcome evaluation, and basic research. Additionally, this review critically examines the limitations of current research on MMD and explores potential future directions, aiming to offer valuable insights and guidance on MMD.

## 1 Introduction

Moyamoya disease (MMD) is a rare, chronic progressive cerebrovascular disorder characterized by the stenosis of the terminal segments of the internal carotid arteries, accompanied by the formation of a “puff-of-smoke” vascular network at the skull base (1, 2). MMD is prevalent in East Asian countries such as Japan and China, and its incidence is increasing (3). MMD tends to affect younger individuals, demonstrating a characteristic bimodal age distribution with incidence peaks around ages of 10 and 40 (4). Clinical presentations are diverse and may include transient ischemic attacks, ischemic stroke, hemorrhagic stroke, movement disorders, headaches, cognitive impairment, and seizures (5). The high incidence of stroke and early cognitive dysfunction observed in patients with MMD may pose significant risks and burdens. Specifically, stroke events can result in severe sequelae such as paralysis and aphasia, and may even be life-threatening. Without timely intervention, early cognitive impairment can progress to dementia, ultimately leading to a complete loss of independent living ability. Moreover, the high costs of treatment and rehabilitation, the need for long-term professional care, and the loss of patients’ working capacity impose significant financial and caregiving burdens on families.

The pathogenesis of MMD remains unclear. Current research suggests that its pathogenesis is multifactorial in origin, involving the interplay of genetic susceptibility, inflammatory and immune responses, and environmental influences (6). Evidence also indicates that vascular reconstructive surgery is an effective treatment, with timely intervention can substantially improve patient outcomes (7). Thus, early and accurate diagnosis and intervention are crucial for MMD patients.

Currently, the diagnosis of MMD relies primarily on imaging examinations. As the gold standard for MMD diagnosis, digital subtraction angiography (DSA) clearly demonstrates the degree of intracranial vascular stenosis and the formation of abnormal collateral circulation. In addition to DSA, magnetic resonance imaging (MRI) and magnetic resonance angiography (MRA) also play significant roles in detecting the condition (8, 9). However, current technologies have notable limitations. Diagnosis often depends on the clinician’s experience, and complex cases may carry a risk of misdiagnosis or missed diagnosis (10, 11). Persistent challenges remain in disease prediction, risk stratification, and therapeutic decision-making.

In recent years, artificial intelligence (AI) technology has advanced rapidly. AI simulates human cognitive functions—including learning, reasoning, and decision-making—through computational algorithms, enabling systematic extraction of information from complex datasets, identification of associative relationships, and automation of sophisticated tasks traditionally requiring human intellectual involvement (12, 13). In medical applications, AI has been integrated across various aspects, including disease screening, diagnostic decision-making, and prognosis prediction (14, 15). Core AI technologies include machine learning (ML), deep learning (DL), natural language processing, and computer vision. Among these, ML and DL are most widely used in medical research. Machine learning, a subset of AI, allows systems to learn autonomously from data and improve through experience. Common ML models include K-Nearest Neighbors (KNN), Random Forest (RF), and Support Vector Machine (SVM) (16). Deep learning, a branch of ML, utilizes multi-layer neural networks to simulate the human decision-making processes and automatically learn complex data features (17). Prominent DL models include Convolutional Neural Networks (CNNs) and Recurrent Neural Networks (RNNs). Recently, researchers have increasingly applied AI to MMD studies. More article details can be found in Table 1.

**Table 1:** Applications of artificial intelligence in moyamoya disease.

References	Application	Methodology	Multi center	Data size	Data type
Kim et al. (19)	Diagnosis	CNN	Yes	753	Skull radiograph
Hao et al. (20)	Diagnosis	CNN	No	80	DSA
Lei et al. (21)	Diagnosis risk factors	ResNet-152; MV-CNN	No	960	DSA
Hu et al. (22)	Diagnosis	3D CNN	No	630	DSA
Akiyama et al. (24)	Diagnosis and differential diagnosis	VGG16	No	210	T2WI
Lu et al. (25)	Diagnosis and differential diagnosis	DenseNet121, ResNet50, SENet154, SEResNet50, SEResNext50	No	660	TOF-MRA
Hong et al. (28)	Diagnosis	ResNeXt50	No	1,683	Retinal photographs
Gao et al. (29)	Diagnosis	SVM, RF, XGBoost	No	128	Cerebral oxygen saturation signals
Yin et al. (32)	Risk factors	ResNet18	No	116	MRA
Sato et al. (33)	Risk factors	RF, gradient boosting,	No	301	DSA
Chen et al. (35)	Risk factors	XGBoost, MLR, SVM, RF, NB	No	790	Clinical data
Chen et al. (34)	Risk factors	LR, SVM, CatBoost, RF, LGBM	No	945	Clinical data
Tompkinset al (39)	Risk factors	ANNSoft, LS, RF, SVM, DT	No	1,202	T1WI
Weng et al. (40)	Risk factors	SR	Yes	187	^18^F-FDG PET
Lei et al. (41)	Risk factors	SR, FC	No	105	rs-fMRI
Zhang et al. (44)	Treatment	3D CA-ResNet; 3D VGG; 3D ResNet; 3D SE-ResNet	No	100	TOF-MRA
Xu et al. (45)	Treatment	P3D ResNet	No	406	DSA
Hou et al. (46)	Treatment	DLRS	Yes	235	rs-fMRI
Wang et al. (47)	Treatment	SVM	No	39	Hypergraph of rs-fMRI
Wang et al. (48)	Treatment	ML	No	118	MRI
Li et al. (49)	Treatment	Delta-radiomics models	No	53	DSA,CTP
Sangwon et al. (50)	Treatment	RF, LR, XGBoost	No	39	Hemodynamic parameter
Gao et al. (54)	Treatment	CPM	No	32	Resting-state functional connectivity
Fuse et al. (57)	Treatment	SVM, RF, LGBM	Yes	512	Perioperative clinical data
Weng et al. (58)	Basic research	NN, AdaBoost, RR, kNN, NB	Yes	288	Serum Metabolic Fingerprints
Xu et al. (59)	Basic research	SVM-RFE, RF, LASSO	Yes	266	Genes
Han et al. (60)	Basic research	SVM-RFE, LASSO	Yes	-	Genes

CNN, Convolutional Neural Network; MV-CNN, multi-view conventional neural network algorithm; 3D CNN,3D convolutional neural network; SVM, Support Vector Machine; SVM-RFE, support vector machine-recursive feature elimination; RF, Random Forest; LASSO, least absolute shrinkage and selection operator; NN, Neural Network; AdaBoost, Adaptive Boosting; RR, Ridge Regression; KNN, K-Nearest Neighbor; NB, Naive Bayes; MLP, Multilayer Perceptron; LR, Logistic Regression; LGBM, Light Gradient Boosting Machine; ANNSoft, Artificial Neural Network; LS, Least Squares Prediction using Bootstrap Aggregation; DT, Decision Tree; SR, Sparse Representation; FC, Functional Connectivity; P3D ResNet, Pseudo-Three-Dimensional Residual Network; DLRS, deep-learning resting-state; ML, Machine Learning; CPM, connectome-based predictive modeling.

**Figure 1:**
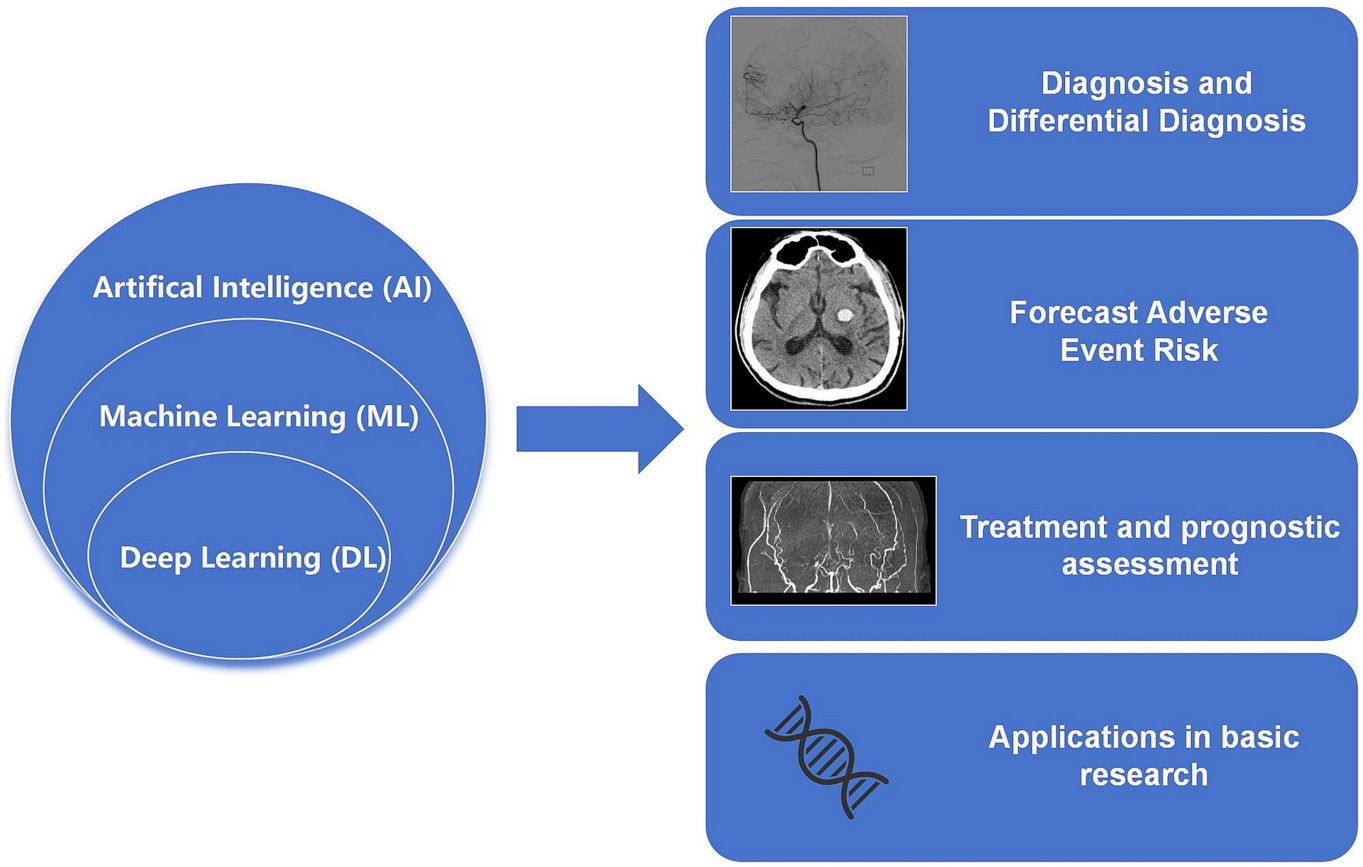
Summary of the main aspects of this review.

This review summarizes the advancements of AI in the diagnosis of MMD, prediction of risk factors, treatment planning, and basic research. It also highlights the limitations of current research in this field and provides recommendations and perspectives for future studies (Figure 1).

## 2 Applications of artificial intelligence in moyamoya disease

### 2.1 Applications of artificial intelligence in the diagnosis of moyamoya disease

Imaging examination is a primary method for the diagnosis of MMD. Compared to manual image interpretation, AI can detect subtle features (e.g., texture patterns, contrast, and coarseness) in images that are imperceptible to the human eye. It then uses algorithms to classify and predict based on these features, enabling rapid and accurate diagnosis (18). The application of AI models has improved the accuracy and efficiency of MMD detection. When integrated into clinical practice, these models have the potential to significantly reduce radiologists’ workload.

Kim et al. (19) established a cohort comprising 345 MMD patients and 408 healthy controls, and trained a CNN-based binary classification model using plain skull images as input. The model demonstrated strong discriminative performance, achieving a diagnostic accuracy of 0.84 and an area under the receiver operating characteristic curve (AUROC) of 0.91 for MMD identification. Importantly, this study first demonstrated that deep learning can extract MMD-related features from plain skull images, which have traditionally been considered of limited diagnostic value, thereby challenging the conventional belief that plain radiographs are ineffective for detecting cerebrovascular abnormalities.

AI-based diagnostic models utilizing DSA have demonstrated distinct technical advantages in the accurate identification of MMD. Hao et al. (20) explored preprocessing optimization strategies for DSA images through noise removal, image enhancement, and normalization. These approaches substantially improved the diagnostic performance of the CNN model, with the AUC increasing from 0.78 to 0.98, highlighting the critical role of data preprocessing in enhancing model effectiveness. Although the study was limited by a small sample size (*n* = 80), its methodological innovation provides an important reference for future research. Lei et al. (21) utilized a large volume of high-quality raw data and developed an end-to-end learning framework based on a deep residual network (ResNet-152), integrating feature extraction and disease classification into a single step. The model extracted features and identified unilateral moyamoya-related vascular lesions directly from raw DSA images. It achieved an AUC of 0.99, with corresponding accuracy, sensitivity, and specificity of 0.97, 0.96, and 0.98, respectively. However, the models used in the above studies were limited to the analysis of static images. DSA not only captures static morphological features of blood vessels but also reflects the dynamic contrast-filling process within the vasculature. To address this, Hu et al. (22) proposed a deep learning architecture combining 3D CNN and Bidirectional Convolutional Gated Recurrent Unit (BiConvGRU). This model simultaneously analyzes the spatiotemporal features of DSA sequences, capturing both the spatial distribution of vascular structures and the temporal evolution patterns of contrast agent filling. This approach significantly enhances the detection performance for MMD.

Conventional MRI and MRA, as non-invasive imaging modalities, demonstrate significant clinical value in the screening and differential diagnosis of MMD (23). Recent advancements in deep learning-based medical image analysis have significantly progressed in this field. Akiyama et al. (24) developed an automated method combining conventional T2-weighted imaging (T2WI) with the VGG16 model to differentiate MMD, atherosclerotic cerebrovascular disease, and normal vasculature. Their analysis focused on three anatomical levels—the basal cistern, basal ganglia, and centrum semiovale—achieving classification accuracies of 0.93, 0.85, and 0.89, respectively. These results suggest that integrating conventional MRI sequences with deep learning techniques can effectively distinguish MMD from other cerebrovascular disorders, providing a cost-efficient strategy for early-stage screening. In the analysis of MRA images, Lu et al. (25) optimized five common deep learning classification networks based on CNN: DenseNet-121, ResNet-50, SENet-154, SE-ResNet-50, and SE-ResNeXt-50. Among these, the DenseNet-121 model demonstrated superior diagnostic performance for MMD, achieving a diagnostic accuracy of 0.86 and an AUC of 0.97. Notably, both studies employed Gradient-weighted Class Activation Mapping (Grad-CAM) for visualization. This technique generates localization maps by extracting the gradients of the target concept from the final convolutional layer, visually highlighting anatomical regions critical for diagnostic decisions (26). This technique not only enhances the interpretability of deep learning models but also supports clinicians in understanding AI-based diagnostic decisions.

In addition to imaging examinations, AI has also been widely integrated with diverse diagnostic techniques for the assessment of MMD. Based on the mechanism that involvement of the internal carotid artery siphon segment in MMD can lead to secondary abnormalities in central retinal artery perfusion (27), Hong et al. (28) innovatively combined retinal vascular morphological assessment with AI technology to develop a diagnostic system based on the ResNeXt50 model. By capturing subtle morphological changes in retinal vessels, the model enabled automated staging of MMD, achieving an AUC of 0.94. This study was the first to validate the feasibility of retinal examination as a noninvasive grading tool, opening a new dimension for dynamic disease monitoring. Near-infrared spectroscopy (NIRS) enables dynamic monitoring of changes in oxyhemoglobin concentration within brain tissue, providing an effective means of assessing cerebral hemodynamics and oxygen metabolism in MMD patients. Researchers employed three algorithms—SVM, RF, and extreme gradient boosting (XGBoost)—to construct MMD screening models based on NIRS monitoring signals of varying durations. The study found that when based on 20-min monitoring data, the SVM, RF, and XGBoost models achieved accuracies of 0.87, 0.85, and 0.85, respectively. Notably, even when the signal acquisition time was reduced to 5 min, the models maintained high accuracy levels of 0.88, 0.88, and 0.84, respectively (29). These findings indicate that algorithmic optimization can overcome the traditional reliance of NIRS technology on prolonged monitoring. By shortening the detection duration without compromising diagnostic performance, the practical value of NIRS in rapid MMD screening scenarios is significantly enhanced.

### 2.2 Applications of artificial intelligence in predicting the risk of adverse events in moyamoya disease

MMD presents a complex clinical course, primarily characterized by ischemic and hemorrhagic stroke events, as well as cognitive impairment (30). In this field, AI can integrate multimodal clinical and imaging data to systematically identify risk factors strongly associated with adverse events and develop predictive models. These models have the potential to assist clinicians in making timely therapeutic decisions and improving patient outcomes.

#### 2.2.1 Analysis of risk factors for stroke

Stroke is one of the most common clinical manifestations of MMD, characterized by high recurrence and disability rates, with severe cases posing life-threatening risks (2, 31). The occurrence of MMD-related stroke involves multiple risk factors, and early identification along with precise intervention is crucial for improving patient outcomes.

Lei et al. (21) developed a multi-view convolutional neural network (MV-CNN-C) model that integrated demographic factors, such as age, sex, and hemorrhagic risk factors (e.g., hypertension, smoking), along with DSA imaging features of MMD patients. The model achieved 0.90 accuracy in predicting unilateral hemorrhage risk. AI enhances risk prediction performance by combining imaging features with clinical data. Yin et al. (32) constructed a ResNet18 model based on transfer learning to perform quantitative analysis of MRA images at the basal cistern, basal ganglia, and centrum semiovale. The study identified the basal cistern (accuracy 93.3%) and basal ganglia (accuracy 91.5%) as key regions for distinguishing hemorrhagic MMD. Through Grad-CAM visualization, the model highlighted abnormal structures within the deep white matter and periventricular collateral vessels, indicating that hemodynamic disturbances in these regions are key triggers of hemorrhagic events. Notably, the periventricular anastomotic (PA) network, a compensatory pathological feature of MMD, is significantly associated with future hemorrhagic events due to the formation of aneurysms within this vascular network. A previous study (33) employed Gradient Boosting and RF models to identify three major risk factors for PA aneurysm: PA score, initial modified Rankin Scale (mRS) score, and age. These findings provide a foundation for clinical monitoring and potential interventions aimed at reducing the risk of hemorrhagic stroke. Chen et al. (34) utilized XGBoost, logistic regression (LR), and SVM models to systematically identify independent risk factors for hemorrhagic stroke in MMD, including advanced Suzuki stage, presence of aneurysms, rural residence, frequent hospitalization history, and age at onset. Furthermore, LR, SVM, CatBoost, RF, and LightGBM models were employed to predict stroke recurrence in adult MMD patients. The analysis revealed that advanced Suzuki stages, younger age (18–44 years), absence of surgical treatment, and the presence of aneurysms were closely associated with stroke recurrence (35). Compared with traditional statistical methods, the machine learning models employed by Chen et al. can automatically detect and leverage interaction effects and nonlinear relationships among relevant factors (17, 36), offering greater adaptability and flexibility. As a result, the predictive performance of these models is more objective, accurate, and reliable.

#### 2.2.2 Cognitive impairment risk factor analysis

Patients with MMD may exhibit cognitive decline in the early stages of the disease (2, 37). This phenomenon is strongly associated with progressive neurostructural and functional alterations that substantially impair quality of life. Based on multimodal imaging, AI can integrate multidimensional information from structural, metabolic, and functional networks, offering an innovative solution for predicting the risk of cognitive impairment. At the structural level, cortical thickness alterations in MMD correlate with memory and executive function deficits (38). Tompkins et al. (39) utilized machine learning models such as SVM and RF to quantitatively identify cortical thickness differences between MMD patients and healthy controls based on structural T1-weighted MRI. This study confirmed that AI can predict the risk of cognitive decline through structural feature analysis. In addition to structural features, researchers have further revealed the impact of metabolic and functional network dynamics on cognitive impairment. One study implemented a sparse representation classifier based on 18F-FDG PET to reveal specific cerebral metabolic patterns in patients with cognitive impairment. This method utilized an adaptive feature filter to select key features (quantifying importance through absolute values of representation coefficients), combined orthogonalization for redundancy removal with structural preservation constraints to optimize sample architecture, and performs classification prediction via reconstruction residual minimization. The approach significantly improves the sensitivity and specificity of abnormal metabolic pattern recognition, providing novel perspectives for early risk prediction (40). Another investigation (41) constructed a dynamic functional connectivity (FC) network integrated with sparse representation classifiers to analyze functional magnetic resonance imaging (fMRI) data, enabling early detection of vascular cognitive impairment (VCI) and identification of clinically significant risk factors associated with VCI pathogenesis. The integration of multimodal imaging and AI enables comprehensive risk assessment of cognitive impairment through multilevel analyses spanning structural, metabolic, and dynamic network dimensions, providing critical evidence for early clinical intervention.

### 2.3 Application of artificial intelligence in the treatment of moyamoya disease

As the pathogenesis of MMD remains unclear, no drugs are currently available to reverse the pathological progression of the disease. At present, cerebral revascularization surgery is considered the primary effective treatment for MMD (42, 43). The integration of AI in MMD not only provides decision support for surgical strategy planning but also enables systematic evaluation of postoperative efficacy and prediction of adverse event risks, providing innovative directions for precision therapy.

#### 2.3.1 Pre-treatment assessment

Precise preoperative evaluation is a crucial element in developing individualized surgical strategies for cerebral revascularization. AI facilitates comprehensive pre-treatment assessment through the analysis of multimodal data. In vascular structure evaluation, Zhang et al. (44) developed a three-dimensional coordinate attention residual network (3D CA-ResNet) deep learning model. This architecture extends network depth through a modified 3D ResNet framework while integrating an enhanced 3D coordinate attention (CA) module into the non-identity branches of residual blocks. The CA module specifically addresses accuracy degradation during training caused by increased network depth. When applied to detect vascular stenosis regions in time-of-flight magnetic resonance angiography (TOF-MRA) images of MMD patients, the model achieved an AUC of 0.94, providing critical guidance for precise surgical target selection. To further determine the optimal timing for surgical intervention, Xu et al. (45) proposed a pseudo-three-dimensional residual network (P3D), which enabled automated classification of MMD disease progression stages and established a quantitative framework for dynamically assessing intervention timing. For cerebral hemodynamic assessment, researchers developed a deep learning network capable of generating cerebral hemodynamic functional maps based on resting-state functional MRI (rs-fMRI). The model enabled reproducible mapping of cerebrovascular reactivity (CVR) and bolus arrival time (BAT). This achieved a comprehensive evaluation of hemodynamics in patients with MMD (46). In addition, traditional surgical evaluations often overlook neuropsychological factors. Wang et al. (47) innovatively proposed a subtype classification of MMD based on cognitive and emotional features—high depression–high anxiety–low cognition, low depression–low anxiety–high cognition, and low depression–low anxiety–low cognition. They developed an SVM model that accurately classified these three subtypes using hypergraph features derived from rs-fMRI. This model enables the incorporation of neuropsychological impairment into the surgical risk assessment framework for MMD, providing a multidimensional and quantitative basis for treatment decision-making.

#### 2.3.2 Treatment effect evaluation and prognosis prediction

By integrating imaging features, intraoperative parameters, and clinical data, AI enables a multidimensional evaluation of surgical outcomes in MMD, including hemodynamic improvement, collateral circulation formation, anastomotic patency, and cognitive function recovery. Wang et al. (48) developed the T1ML model based on MRI imaging features of patients who underwent STA–MCA bypass, enabling noninvasive and accurate identification of postoperative collateral vessel formation, with an accuracy of up to 0.80. Li et al. (49) quantitatively evaluated cerebral hemodynamic changes before and after revascularization in MMD patients using CT perfusion and constructed a delta-radiomics model based on changes in the time-to-drain (TTD) parameter to identify postoperative collateral vessel formation, which provided a comprehensive assessment of surgical outcomes. In parallel, researchers have further evaluated intraoperative hemodynamic characteristics. Using three ML models (RF, LR, and XGBoost), they predicted postoperative anastomosis patency by analyzing real-time intraoperative blood flow velocity and volume features obtained via FLOW 800 technology, thereby offering surgeons with real-time decision support (50). Multiple studies have demonstrated that early surgical intervention improves cognitive impairment in patients with MMD (51–53). Therefore, cognitive function changes constitute a critical component of postoperative outcome evaluation. Connectome-based predictive modeling (CPM) was employed by Gao et al. (54) to analyze preoperative resting-state functional connectivity features and predict the degree of improvement in information processing speed after surgery, offering a novel perspective for evaluating postoperative cognitive recovery.

Although surgical intervention effectively reduces the risk of cerebrovascular events and improves long-term prognosis in MMD patients, postoperative complications such as cerebral infarction remain a major challenge affecting treatment outcomes (55, 56). Fuse et al. (57) applied machine learning models, including SVM, RF, and Light Gradient Boosting Machine (LGBM), to analyze perioperative clinical data and predict the incidence of postoperative cerebral infarction. To enhance model interpretability, the study incorporated SHAP (Shapley Additive Explanations) analysis, a technique grounded in Shapley value theory from game theory, which quantifies the contribution of each feature in individual cases to reveal the decision-making logic of the AI model. SHAP analysis identified pentachlorobenzene ether, PCA, infarction at the surgical site, and the presence of infarction as risk factors for postoperative cerebral infarction. These findings may help optimize perioperative management and guide postoperative care, thereby improving long-term prognosis and quality of life in patients with MMD.

### 2.4 Applications of artificial intelligence in the basic research of moyamoya disease

In the field of basic research on MMD, AI is driving progress across multiple dimensions, including the elucidation of disease mechanisms, the discovery of biomarkers, and the innovation of therapeutic strategies. Weng et al. (58) applied various machine learning algorithms, including neural networks (NN) and adaptive boosting (AdaBoost), to analyze serum metabolic fingerprints from 144 MMD patients and 144 healthy individuals, achieving accurate differentiation between the two groups (AUC = 0.95, 95% CI: 0.911–1.000). Furthermore, they employed the Integrated Gradients (IG) method to quantify the contribution of metabolic signals to model predictions. By combining fold change (FC) analysis and t-tests, six key metabolites associated with MMD were identified, providing a novel metabolism-based approach for the noninvasive diagnosis of MMD. At the genetic level, Xu et al. (59) identified 266 key MMD-related genes using weighted gene co-expression network analysis (WGCNA), followed by KEGG and GO enrichment analyses to construct a protein–protein interaction (PPI) network. Three machine learning algorithms, including support vector machine-recursive feature elimination (SVM-RFE), RF, and LASSO, were subsequently applied for cross-validation, resulting in the identification of four high-confidence diagnostic biomarkers (ACAN, FREM1, TOP2A, UCHL1). These genes were found to be functionally associated with specific immune cell subpopulations. Moreover, another study integrated MMD-related data from the Gene Expression Omnibus (GEO) and GeneCards databases to systematically identify key genes involved in the oxidative phosphorylation (OXPHOS) pathway. Using LR and SVM-RFE algorithms, four core genes most strongly associated with MMD pathogenesis (CSK, NARS2, PTPN6, SMAD2) were selected from the OXPHOS gene set. Gene Set Enrichment Analysis (GSEA) provided insights into immune cell infiltration and the vascular microenvironment related to these genes, ultimately establishing a connection between the four key OXPHOS-related genes and the pathogenesis of MMD (60). Collectively, AI-driven identification of MMD genetic biomarkers not only provides novel targets for early diagnosis but also enables therapeutic strategies targeting immune microenvironment modulation.

## 3 Limitations and future perspectives

### 3.1 Limitations

Recent studies demonstrate that AI demonstrates significant potential in the diagnosis, risk factor analysis, and treatment evaluation of MMD. However, there are still several limitations in this area. Current AI model development requires large-scale, high-quality data. However, MMD is a rare disease with low incidence, and the integration of multicenter data remains challenging. As a result, the diagnostic accuracy of current models falls short of clinical requirements. Furthermore, research on AI applications in MMD is predominantly based on single-center studies. Variability in data formats, imaging equipment, and diagnostic standards across medical institutions limits the generalizability of AI models. The high computational demands of current AI systems are often impractical in many clinical settings. In addition, the lack of clearly defined regulations regarding patient privacy protection and medical liability further complicates clinical implementation. These challenges collectively pose significant barriers to the widespread adoption of AI in MMD clinical practice. While some AI models have demonstrated strong predictive performance, they often lack transparency in their algorithms and decision-making processes, resulting in limited interpretability. Consequently, clinicians may question the reliability of model predictions and hesitate to apply them in real-world medical decision-making.

### 3.2 Future perspectives

In the future, the application of AI in the field of MMD holds significant potential for further development. By establishing multi-center collaborations and integrating MMD case data across different regions, disease stages, and types of complications, it is possible to effectively address challenges such as limited sample size, high data heterogeneity, and inconsistent data quality. Multimodal data research that integrates imaging, genomics, and clinical information can further enhance AI models, enabling a more comprehensive evaluation of the pathophysiological mechanisms, clinical manifestations, and risk factors associated with MMD. To address the limited interpretability of current AI models and algorithms, it is necessary to develop more transparent models, incorporating visualization techniques such as Grad-CAM and SHAP to elucidate the decision-making process and the underlying rationale. This will enhance the transparency of AI models, facilitating their adoption in clinical practice. With the continuous advancement of the technology, AI can serve as a powerful tool for the diagnosis and treatment of MMD, enabling early diagnosis and timely treatment of MMD patients, thus significantly improving the prognosis of MMD patients.

## 4 Conclusion

This article reviews recent developments in the application of AI in the diagnosis and differential diagnosis, risk factor analysis, treatment, and basic research of MMD, highlighting the potential of AI in this field. Although significant challenges remain, the continuous advancement of AI technology and deeper exploration in the field are expected to revolutionize clinical practices in the diagnosis and treatment of MMD, offering patients more accurate and effective medical care.
